# Drug Discovery for Snakebite Envenoming: Wanderings Amid Historical Antidotes and Emerging Therapeutic Opportunities

**DOI:** 10.1002/cmdc.70478

**Published:** 2026-10-31

**Authors:** Elisa Bianconi, Emidio Camaioni, Paulina Oszajca, Antonio Macchiarulo

**Affiliations:** ^1^ Department of Pharmaceutical Sciences University of Perugia Perugia Italy; ^2^ Swiss Society for the History of Pharmacy Zurich Switzerland

**Keywords:** drug repurposing, historical antidotes, rosmarinic acid, snakebite, varespladib

## Abstract

Snakebite envenoming is acknowledged as neglected tropical disease affecting populations of regions with limited access to healthcare systems. However, it had posed a major threat to human health since classical antiquity, when plant‐ and animal‐based remedies were extensively used as antidotes to counteract bites by venomous snakes. Although the provision of safe and effective treatments by 2030 is among the strategic objectives of the World Health Organization, clinically approved therapies currently available remain animal‐derived polyclonal antibodies, which suffer from significant limitations, including high production costs, limited cross‐species efficacy, and risk of adverse immune reactions. In this review, we survey some historical antidotes used to treat snakebite envenoming in classical antiquity and early modern Europe such as theriac, mithridatum, Orvietan and Mattioli's scorpion oil. We then discuss natural compounds that, while found also as active ingredients in medical plants used for the preparation of the historical antidotes, over the last decades have inspired research efforts aimed at characterizing their evidence‐based antivenom activity with the aim of offering potential alternatives to biologic‐based therapies. Finally, we outline emerging therapeutic opportunities that, arising from drug repurposing efforts, may pave the way for the development of novel, broadly effective therapies against multiple venomous snake species.

## Graphical abstract

In this review, we provide an overview of selected historical antidotes against snakebite envenomation used in early modern Europe. Then, the evidence‐based antivenom activity of some natural compounds is discussed as resulting from more than two decades of research studies. Finally, drug repurposing efforts are outlined that may pave the way to the development of new effective therapies against multiple venomous snake species.

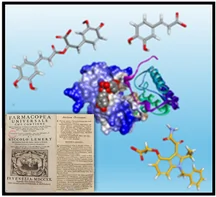

## 1 Introduction

Snakebite envenoming has been a common and critical health threat in ancient times. Yet, it still represents a global health issue, impacting nowadays rural populations with very limited access to education and health care systems. According to the World Health Organization (WHO), snakebites account for 5.4 million people worldwide each year, with 2.7 million envenoming and 83,000–138,000 yearly deaths [1]. The provision of safe and effective treatments is amid the strategic objectives settled by the WHO to reduce mortality and disability of snakebite envenomation by 50% before 2030. Considering the complexity and high variability of snake venom toxins, which is further complicated by inter‐ and intraspecies differences [2], this task remains challenging for drug hunters since the mists of time.

Four major toxin families are the most abundant and conserved in the composition of medically relevant snake venoms, thereby constituting key targets for the design and development of therapeutics against snake envenoming and associated syndromes [3]. Phospholipases A2 (PLA2s) are the most abundant toxins in venoms of viper and elapid snakes and have neurotoxic, cytotoxic, and hemotoxic actions, depending on the affected cell type [4, 5]. They are divided into two classes: catalytically active and catalytically inactive (or poorly active) PLA2s, with the latter also termed PLA2‐like and commonly possessing an Asp49 by Lys49 (or Arg49, Ser49, Asn49, and Gln49) substitution [6]. While members of the first class (PLA2s) exert disruption of cellular membranes by binding to specific membrane protein receptors and catalyzing the hydrolysis of phospholipids, PLA2s‐like toxins dock to membrane phospholipid sites by electrostatic interactions and induce a disrupting perturbation of the plasma membrane without hydrolyzing phospholipids [7, 8, 9]. Because of plasma membrane damage, the affected cells, such as presynaptic neurons, myocytes, dermal, and vascular cell systems, become more permeable to calcium influx, which leads to cytoskeleton alteration, mitochondrial dysfunction, and necrosis. Some secreted PLA2s of elapids and viperids also exert antiplatelet aggregation and anticoagulant effects by interacting with and inhibiting platelet receptors and coagulation factors [10, 11].

Alongside PLA2s, snake venom metalloproteases (SVMPs) and snake venom serine proteases (SVSPs) are other classes of toxins which are abundant in the venom of viperid snakes and contribute to local tissue damage and systemic dysfunctions [12, 13, 14]. Depending on their structural architecture, SVMPs are grouped into three classes of toxins (P_I_‐P_III_), reflecting their content of functional domains. While the aminoacidic sequence of P_I_ toxins folds in a single catalytic metalloproteinase domain, P_II_ toxins are composed of a second disintegrin‐like domain that can be cleaved and released, and members of the P_III_ group contain an additional cysteine‐rich domain. SVMPs catalyze the hydrolysis of components of the extracellular matrix (ECM), leading to basal membrane damage and disruption of cell–cell junctions with a weakening of microvascular integrity [12, 15]. Notably, these events facilitate the diffusion of venom toxins, bestowing to SVMPs a key role in promoting snake venom toxicological effects at the systemic level [16]. With this regard, the systemic spreading of P_II_ and P_III_ SVMPs, which contain additional domains binding to target membrane proteins in vascular and tissue cells such as renal glomeruli, leads to potent hemorrhagic activity, which reduces blood perfusion to tissues, generating ischemic events and acute kidney injury [17, 18, 19]. SVSPs are monomeric glycoproteins containing a conserved catalytic site, characterized by a catalytic triad comprising a reactive serine, a histidine, and an aspartic acid. As proteolytic enzymes, SVSPs degrade proteins involved in blood coagulation, thereby causing the hemostatic alterations observed following snakebite envenomation [14].

In addition to PLA2s, SVMPs, and SVSPs, snake venoms also contain families of nonenzymatic proteins, among which three‐finger toxins (3FTX) stand out for their potent neurotoxic and cytotoxic activity. They are mostly found in the venom of elapids and share a common fold composed of a central core with three protruding loops [20]. It has been proposed that cytotoxic 3FTXs bind to membrane phospholipids, perturbing the integrity of the plasma membrane by pore formation [21]. They may also be internalized into the cytosol, leading to cell damage and death by the activation of still elusive signaling pathways [22]. α‐Neurotoxins are members of the 3FTX protein family, interacting with ionotropic nicotinic receptors on postsynaptic neuromuscular junctions. Acting as curare mimetic, they induce flaccid paralysis [23]. Fasciculins are another subgroup of neurotoxic 3FTXs binding to acetylcholinesterase and inhibiting the hydrolysis of acetylcholine [24].

Other less prevalent enzymatic toxins are part of the complex composition of snake venom mixture, such as hyaluronidases and L‐amino acid oxidase (LAAO), to name a few [5, 25]. Notably, some cytotoxic 3FTxs and PLA2s are also able to self‐aggregate, forming homo‐ or hetero‐oligomerization complexes that potentiate the cytotoxic effects associated with the relative single entities [26, 27, 28, 29, 30].

The challenge of treating snakebite envenomation has been documented since classical antiquity, with different plant‐ and animal‐based remedies that have been used as antidotes for the treatment of systemic and local toxicological effects. Alexander the Great encountered venomous snakes during his campaign in India [31]. According to *Bibliotheca Historica* of Diodorus Siculus, which describes Alexander's conquests, ancient Hindu doctors already possessed some knowledge of toxicology (*agadatantra*), preparing antidotes by decocting decomposed snakes [32]. One of the most renowned Indian surgeons, Sushruta (first millennium BC), described the diagnosis and treatment of snakebite victims in his *Sushruta Samhita*, a foundational text of Ayurveda [33]. In the Hellenistic world, the Alexandrian school strongly promoted scientific research in iology (*ἰός*‐*λoγία* = venom‐study), namely the study of venoms. Notably, Apollodorus of Tarentum (c. 300 bc), the founding father of Hellenic iology and author of the treatise *Peri Thēriōn* (“On Venomous Animals”), produced one of the most significant early Greek texts on iology. His work served as a main source for the poetic writings of Nicander of Colophon (c. 130 bc), particularly the *Thēriaká* and *Alexiphármaka*. The works of Nicander focus on venomous animals, especially snakes, and the symptoms of their bites, as well as antidotes and treatments [34]. He provides vivid descriptions of the appearance, habitat, and behavior of these animals, as well as the symptoms caused by their bites and how to identify them based on their physical effects. Nicander's poems are the main surviving sources on classical toxicology and belong to a tradition that also shaped later writers such as Galen and Dioscorides. In the medieval period, Avicenna's *Canon of Medicine* and Maimonides’ *On Poisons and the Protection against Lethal Drugs*, originating from the Arabic‐Islamic medical tradition, exerted a significant influence on the development of toxicological knowledge, especially regarding venomous snakebites [35].

In addition to widely used interventions such as applying a tourniquet, sucking out the venom, and preventing the patient from falling asleep, compound medicines (antidotes) were administered both externally as plasters applied to the wound and internally, primarily dissolved in wine. In Western medical tradition, Galenic antidotes such as theriac and mithridatum were widely used to treat various forms of envenomation, including snakebites. From the 16th century onward, new composite antidotes were introduced by scholars, driven partly due to the difficulty of faithfully reproducing ancient recipes and partly by efforts to improve ancient formulations [36]. These complex preparations, such as Mattioli's scorpion oil or the Orvietan, combined numerous botanical ingredients with small amounts of theriac and/or mithridatum. They gained considerable recognition and were even included in some pharmacopeias. By the 19th century, however, they had fallen out of use and were no longer reported in medical treatises, as the scientific community considered them to lack evidence‐based efficacy, although some continued to be produced in later centuries [37, 38].

Nowadays, the only available therapies for snakebite envenoming are antidotes based on animal‐derived polyclonal antibodies. However, these biological products suffer from major limitations, including high production costs, complex supply chains, poor penetration into peripheral tissues, limited cross‐species efficacy, and a high incidence of adverse reactions. Consequently, the quest for safer and more effective therapies is attracting considerable interest.

Beginning with an overview of selected historical antidotes for snakebite envenomation described in medical treatises of early modern Europe, this review focuses on some natural compounds that have inspired research efforts aimed at characterizing their evidence‐based antivenom activity and small‐molecule‐based strategies emerging from drug repurposing efforts that may revolutionize snakebite treatment in the next future.

## 2 Historical Antidotes

The term theriac remained ambiguous for centuries as it could refer either to a general category of drugs (theriaca) used against animal venom or to a specific compound regarded as both an antidote and a universal remedy (cure‐all) [39, 40]. King Mithridates VI of Pontus (d. 63 bc) famously attempted to immunize himself against venoms and poisons by regularly ingesting small doses of toxins along with an antidote he developed through personal experimentation, later known as mithridatum [41]. The original recipe for Mithridates’ antidote cannot be definitively reconstructed from existing sources [42]. Later formulations contained from 22 to over 54 ingredients, the most renowned being the version attributed to Damocrates, which contained 49 components, including opium, castoreum, and numerous plant‐derived substances and resins [43]. In the first century AD, Nero's court physician Andromachus the Elder reformulated Mithridates’ remedy by replacing minced lizards (*venter scinci*) with venomous snakes (prepared viper troches) and adjusting the quantity of opium, thereby creating the famed theriac [44]. The theriac made from this recipe was known as the great theriac (*theriaca magna*) or the refined theriac (*theriaca fina*). According to Galen, viper flesh rendered theriac both heating and drying, properties believed to help dissolve and excrete harmful substances from the body. Galen considered mithridatum and theriac to have similar powers, though he viewed theriac as more effective specifically against viper bites [44]. Galen likely improved the method of preparing viper flesh through observation of the practices of the Marsi, an Italic population from the Abruzzo region, who were known to consume snakes for immunization purposes and were believed to possess a natural resistance to venom [45]. Generally, ancient physicians believed that the flesh of venomous animals, such as snakes or scorpions, could act as an antidote since these creatures were thought to be venomous yet resistant to their own toxins [36]. In the Middle Ages, major centers to produce various theriacs emerged in the Levant and Egypt, thanks to easy access to highly valued ingredients that were often difficult to obtain in Europe [46]. In the early modern period, European theriac became popular as Renaissance scholars reinterpreted ancient recipes and promoted the idea of a “true theriac” [40]. Theriac prepared in Venice, a major trading center with access to exotic raw materials, was considered a “blockbuster drug” at that time and was traded throughout both the western and eastern Mediterranean [47]. Also, Roman theriac produced in the pharmacy of the Roman Jesuit College had similar fame and, thanks to the extensive network of Jesuit colleges and missions, became known not only throughout Catholic Europe but also in the overseas colonies, reaching them as part of the Jesuits’ medical practice [48]. Although the Galenic tradition dominated in Europe, it also absorbed elements from the Arabic medical tradition. A particularly informative account is provided by the physician Gentile da Foligno (d. 1348), who treated a young man bitten on the ankle by a venomous snake in the countryside near Perugia. Gentile, known primarily for his commentaries on the *Canon of Medicine*, initially administered Galen's great theriac. When no improvement was observed, he additionally employed an Arabic version of theriac described by Haly Abbas, which contained ingredients such as castoreum, cassia wood, citrus seeds, anise seed, pepper, and aristolochia. He also applied other remedies recommended by Maimonides, including powdered emerald and wine infused with citrus seeds, and eventually succeeded in curing the snakebite envenomation symptoms of the young man [49].

During the Renaissance, a new approach to classical texts emerged. Scholars have focused on identifying raw materials in ancient botanical nomenclature and searching for substitutes of those that could not be reliably identified. The most renowned example is the commentary on Dioscorides’ *Materia medica* by the Sienese physician Pier Andrea Mattioli, first published in Italian in 1544 [50]. Translated into Latin in 1554, it became one of the most widely read scientific works of the 16th century. Mattioli also devised several antidotes for snakebites, the most famous being his scorpion oil, described in his commentary on the sixth book of Dioscorides [36]. Mattioli significantly expanded the *Oleum de Scorpionibus comp*. originally formulated by Mesue, which contained only a few ingredients (e.g., *Aristolochia rotunda* and 15 scorpions). Following Antonio Berthioli's instructions, Mattioli's scorpion oil included thirty‐two ingredients, among them an extract of 300 live scorpions, various herbs, roots, gums, theriac, and mithridatum, combined with each other in nine complex stages of production [51]. The oil was considered a miraculous remedy against scorpion venom and bites from other venomous creatures. Berthioli describes the case of a man bitten by a viper on the foot. Despite severe symptoms, including bruising and shortness of breath, he fully recovered within 2 days after the oil was applied to the wound, pulse points, and chest every 3 h [51].

Another remedy developed from theriac and mithridatum was Orvietan. According to La Martinière, author of a treatise on these three antidotes, Orvietan is believed to have originated in Italy in the late 16th century [52]. The story cited by the French author traces the origins of the formulation back to Rome and links it to the apothecary Martin Guerche, who had two apprentices originating from Orvieto. Contemporary scholars link the origins of Orvietan with Orvieto city and Girolamo Ferrante, who was selling this remedy in Paris at the beginning of the 17th century [53]. Unlike theriac and mithridatum, which had an officially sanctioned formula approved by municipal medical authorities, Orvietan was initially sold as a secret recipe by charlatans. A wide range of versions circulated, with each practitioner modifying or “perfecting” the formula according to their own approach [54]. P. Catellani and R. Console analyzed 35 different Orvietan formulas, in which the number of ingredients ranged from 9 to 57 in the most complex versions [55]. Eventually, Orvietan was incorporated into scientific and official pharmaceutical texts, including private pharmacopeias of Schroeder, Bates, Charas, and Lemery (Figure 1) as well as official Pharmacopeias of London, Lyon, Lille, and Paris [54]. While the exact composition varied, most versions included “aged” theriac as a core ingredient, with this dilution likely making the remedy more affordable and accessible to poorer populations.

**FIGURE 1:**
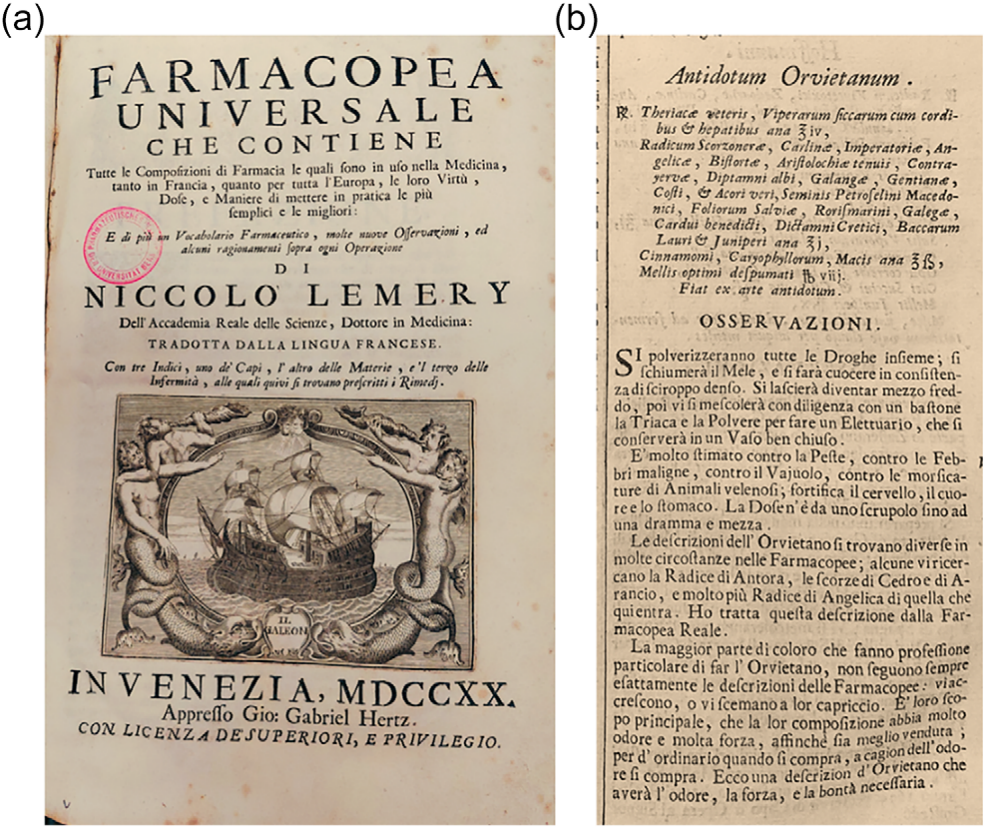
(a) Frontispiece of the Lemery's pharmacopeia, version translated from French to Italian and printed by Hertz in Venice in 1720. (b) The recipe and preparation of the Orvietan (*Antidotum Orvietanum*) and use thereof from Lemery's pharmacopeia [56]. Scanned images from IMG PH Fol 11 P, reproduced with permission from the Historical Library of Swiss Pharmacy (HBSP), Bern (Switzerland).

Although Orvietan was believed to be a product of charlatans, a cheap version of theriac was also distributed by itinerant vendors, who staged dramatic demonstrations to convince the public of their effectiveness. Those theriac hawkers, pejoratively called *triacleur* in French and *Theriakkrämer* in German, appeared at major European markets [57]. Their performances could include spectacular demonstrations involving snakes, toads and other supposedly venomous animals, intented to convince audiences of the efficacy of their remedies. A German Baroque writer depicts a similar performance involving a supposedly venomous toad and theriac, with the animal's death presented as proof of the remedy's efficacy [58].

By the 18th century, theriac preparations, especially those containing viper flesh, came under heavy criticism. John Quincy denounced Venetian treacle for its ineffective ingredients and proposed alternative formulas [59]. Likewise, William Heberden, in his treatise *Antitheriaka*: *An Essay on Mithridatium and Theriaca* (1745), called for the removal of theriac from the official London Pharmacopoeia [60]. Nonetheless, theriac has survived in traditional systems of medicine. In *Unani*, an ancient and traditional Indian medicine which is acknowledged by the WHO, it continues to be used under the name *tiryaq faruq* as a treatment for envenoming, infections, and chronic diseases [61]. This preparation contains 63 ingredients, including *qursifai*, namely dried tablets made from viper flesh [62].

## 3 Emerging Therapeutic Opportunities

Over more than two decades, biological and pharmacological investigations have proved antivenom properties for several plant extracts of different origins [63, 64, 65, 66], very often building upon studies of plants commonly used in folk medicine of India, South, and Central Americas, rather than historical sources of classical antiquity and early modern Europe [67, 68, 69, 70]. Overall, these studies have put the spotlight on natural compounds as viable complementary and promising alternatives to biologic‐based therapies against snakebite envenomation [71, 72]. In the following paragraphs, we focus on the antiophidian activities of selected natural compounds that are commonly found as active ingredients in some medicinal plants used for the preparation of theriac, mithridatum, Orvietan, and scorpion oil (Table 1). Specifically, although a large diversity of metabolites is found in medicinal plants, compounds belonging to the chemical class of polyphenols (**1**, **2**, **4**; Figure 2) are selected and discussed, since functional data and presence of structure‐based evidence are documented in the literature that support a specific antiophidian mechanism of action. Next, drug repurposing strategies are surveyed as current approaches that have led to identify some known drugs (**5**–**9**) as promising small‐molecule therapies for snakebite envenoming.

**TABLE 1:** Selected medicinal plants in the compositions of some recipes of theriac, mithridatum, Orvietan, and scorpion oil found in medical treatises of early modern Europe.

Selected plants described in historical sources	*Theriaca Andromachi senioris* [73]	*Mithridatium Damocratis* [73]	*Orvietanum* [56]	*Oleum de Scorpionibus Matthioli* [36]	Selected compounds^a^
*Aristolochia clematitis/tenius/rotunda*	x	x	x	x	(**2**, **3**) [74]
*Calamintha nepeta*	x	—	—	x	(**1**, **2**, **4**) [75]
*Daucus*	x	x	—	—	(**1**, **2**, **4**) [76, 77]
*Dictamnus creticus* (*Origanum dictamnus*)	x	x	x	—	(**1**, **2**) [78]
*Foeniculum*	x	x	—	—	(**1**, **2**, **4**) [79]
*Gentiana lutea*	x	x	x	x	(**1**, **2**, **4**) [80]
*Petroselinum macedonicum* (*Petroselinum crispum*)	x	x	x	—	(**2**, **4**) [81]
*Rosmarinus officinalis*	—	—	x	—	(**1**, **2**, **4**) [82]
*Salvia officinalis*	—	—	x	—	(**1**, **2**, **4**) [83]
*Teucrium chamaedrys*	x	—	—	x	(**1**, **2**, **4**) [84]
*Teucrium montanum/polium* (*Polium montanum*)	x	x	—	—	(**1**, **2**, **4**) [85]
*Teucrium scordium*	x	x	—	x	(**2**) [86]

a Chemical structures of the selected compounds are reported in Figure 2.

**FIGURE 2:**
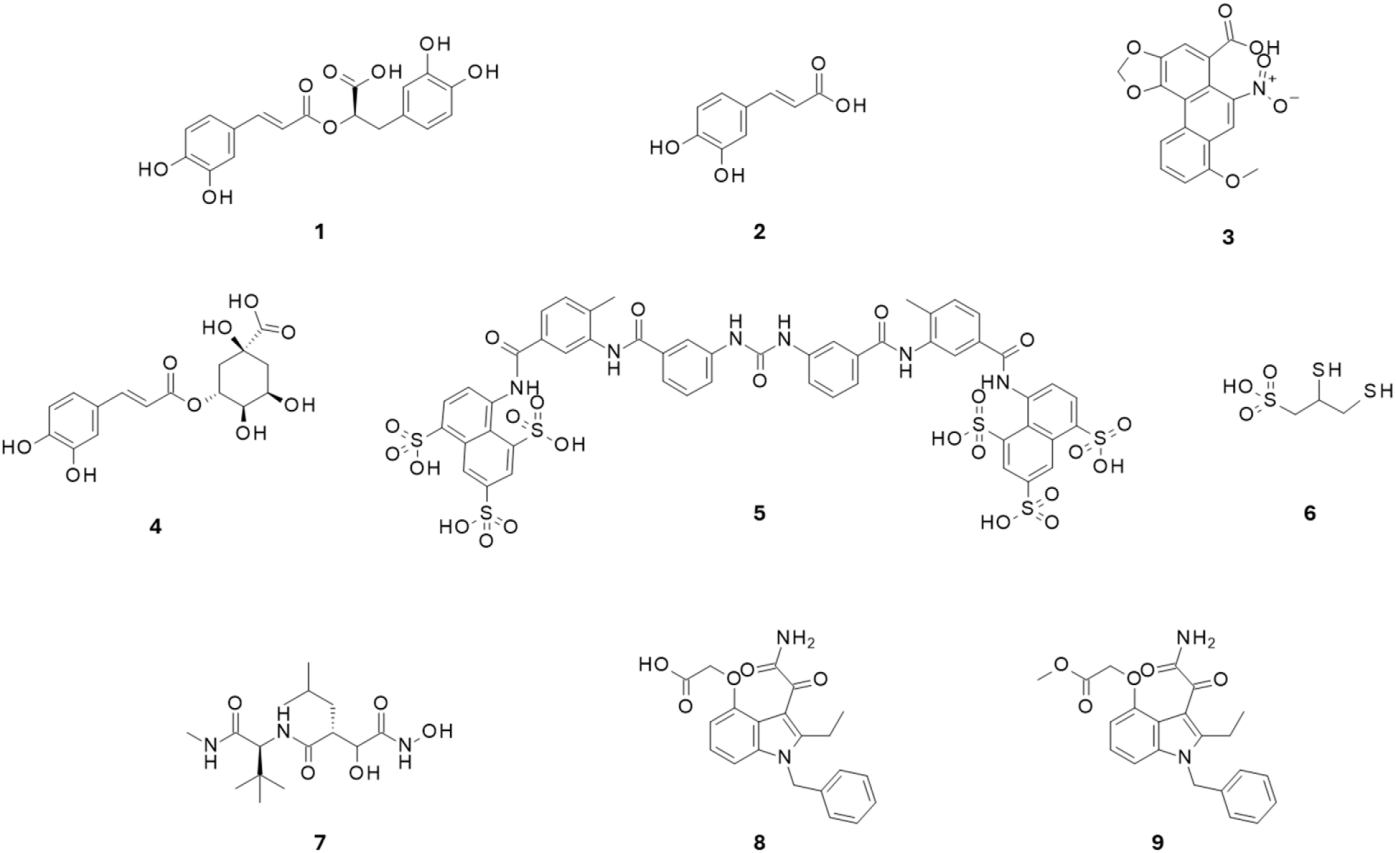
Chemical structures of the selected natural compounds (**1**–**4**) and known drugs (**5**–**9**).

### 3.1 Natural Compounds

Rosmarinic acid (**1**; Figure 2) is among the widely studied natural polyphenols for its multiple pharmacological functions including anti‐inflammatory, neuroprotective, hepatoprotective, antiviral, and anticancer activities [87]. It is mostly found in plants of the *Lamiaceae* and *Boraginaceae* families, such as *Rosmarinus officinalis L*. and *Melissa officinalis L* [88]. Its first isolation and structural characterization were accomplished by two Italian chemists in the mid of ‘900 [89]. The antiophidian activity of rosmarinic acid (**1**) was reported by a research team at the University of Sao Paulo (Brazil) [90]. The authors grounded on the observation that the methanolic extract from *Cordia verbenacea* was able to significantly inhibit the paw edema induced by *Bothrops jararacussu* snake venom in mice and mediated by its main catalytically inactive PLA2‐like (Lys49 Bothrops toxin‐I; BthTX‐I) and catalytically active PLA2 (Asp49 Bothrops toxin‐II, BthTX‐II) enzymes. They identified rosmarinic acid (**1**) as the main active component of the extract, which proved more potent inhibitory effects against edema and myotoxic activity mediated by the isolated BthTX‐I and BthTX‐II than that of the crude venom. In the same work, rosmarinic acid (**1**) was also shown to potentiate the neutralizing activity of the equine‐derived polyvalent antibody therapy against the myotoxic activity of the crude venom and the isolated enzyme BthTX‐I in mice. From a mechanistic point of view, the authors proposed, by molecular modeling studies, that rosmarinic acid (**1**) could bind to BthTX‐I occupying a hydrophobic channel located nearby a conserved membrane disruption site in PLA2‐like toxins of *Bothrops* species [91, 92] while engaging His48 and Lys69 with polar interactions mediated by the caffeic catechol group and the carboxylic group of the compound, respectively. Crystallization studies of rosmarinic acid (**1**) in complex with a homolog PLA2‐like enzyme (PrTX‐I; Figure 3a) isolated from *Bothops pirajai* snake venom supported such scenario, unveiling the molecular recognition pattern and mechanism of inhibition of **1** against this toxin [93]. Specifically, it was found that compound **1** binds to the entrance of the hydrophobic channel adopting a “L”‐shaped conformation with hydrophobic contacts and polar interactions involving one of its aromatic rings and the catechol and carboxylic groups, respectively (Figure 3). As a result of such interactions, rosmarinic acid (**1**) provides steric bulk to the access of phospholipids into the hydrophobic channel of the active dimeric form of PrTX‐I. The docking of membrane phospholipids into the hydrophobic channel has indeed been proposed as the first step of the myotoxic activation mechanism, leading to cell membrane disruption [94, 95]. Blocking this step, rosmarinic acid (**1**) inhibits the cell membrane interaction and destabilization activities of the snake venom toxin.

**FIGURE 3:**
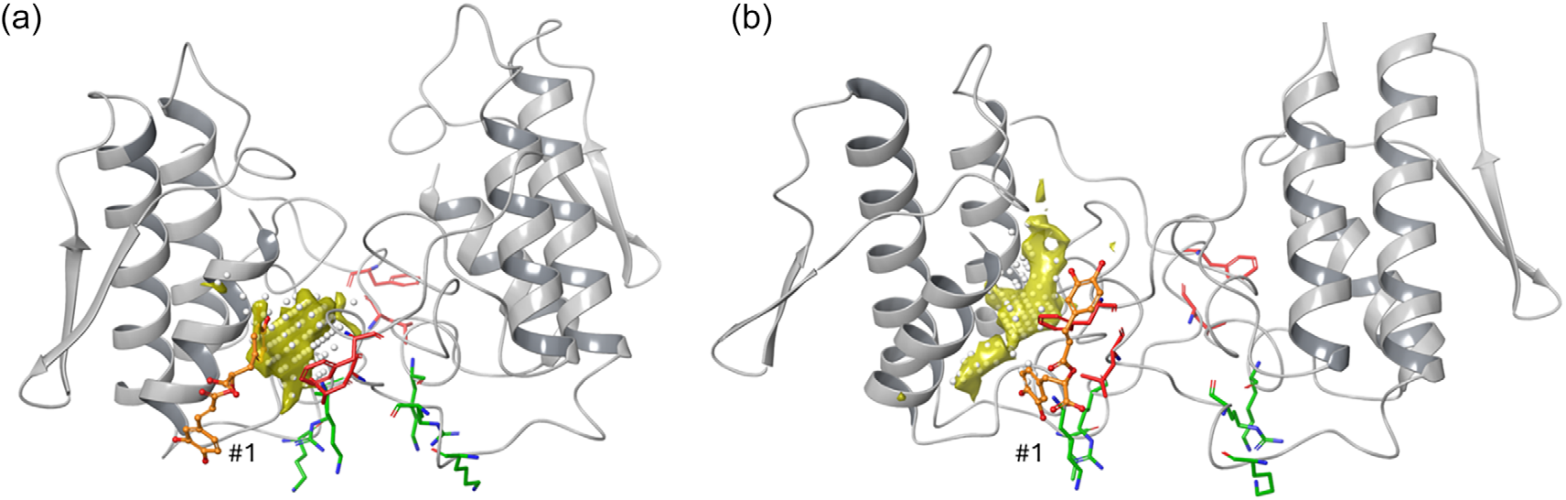
(a) Crystallographic structure of rosmarinic acid (**1**) bound to PrTX‐I dimer (pdb code: 3QNL). (b) Crystallographic structure of rosmarinic acid (**1**) bound to MjTX‐II dimer (pdb code: 6MQD). The hydrophobic channel is mapped with the yellow area using SiteMap (Schrodinger 2025, v.14.6); residues of membrane docking site and disruption site are shown in green‐carbon sticks and red sticks, respectively. Rosmarinic acid is shown in orange‐carbon sticks and the protein in gray ribbons. The ligand binding site is numbered.

In another structure–activity relationship study [96], the inhibitor activity of rosmarinic acid (**1**) against the myotoxic effects of another PLA2‐like toxin (MjTX‐II) isolated from *Bothrops moojeni* venom was investigated. Results show a different mechanism of activation not requiring phospholipids binding to its hydrophobic channel for the alignment of the membrane docking site and membrane‐disrupting site of the toxin to the target cells. While the compound proved to inhibit 87.3% of murine muscle paralysis induced by MjTX‐II after preincubation with the venom toxin, crystallization studies highlighted a different binding mode of rosmarinic acid (**1**) to the protein from that observed in PrTX‐I. In this case, crystallization studies showed that compound **1** adopts an extended conformation, interacting with the dimeric form of MjTX‐II (Figure 3b), occupying a region located at the entrance of the phospholipid hydrophobic channel. Specifically, the catechol group of the phenyl‐lactic fragment engages Asn17 and Ala19 (monomer A) with direct hydrogen bonds, while the phenyl group of the caffeic fragment makes hydrophobic and aromatic contacts with Phe126 (monomer B), and the catechol group engages the carbonyl backbone of Lys69 with a water‐mediated hydrogen bond. In the same study, molecular dynamic simulations supported the stability of this ligand‐binding mode, evidencing a ligand‐induced distortion of the quaternary conformation of the active dimeric form that could also account for the inhibition of the myotoxic activity of MjTX‐II by rosmarinic acid (**1**).

A research team at the Meijo University (Japan) found that the methanolic extract of *Argusia argentea* (Boraginaceae) was able to inhibit the hemorrhagic effects induced by *Trimeresurus flavoviridis* venom and some other snake species (*Crotalus atrox*, *Gloydius blomhoffii*, and *Bitis arietans*) [97, 98]. Rosmarinic acid (**1**) was again found as the main component of the active extract. Specifically, the authors observed that rosmarinic acid (**1**) was able to inhibit the snake venom hemorrhagic effects when injected subcutaneously in the abdomen of mice in combination with the crude venom or with its SVMPs (hemorrhagic toxin‐b, HT‐b; bilitoxin‐2; hemorrhagic toxin‐1, HT‐1; Ac1‐proteinase). This effect was supported by the in vitro antifibrinogenolytic activity of rosmarinic acid (**1**) that proved to inhibit the digestion of human fibrinogen and type IV collagen by snake venom. In further study, the authors leveraged the design and synthesis of compound **1** analogs, including caffeic acid (**2**) and cinnamic acid derivatives, to cast light on the structure–activity relationships for the inhibition of snake venom hemorrhagic activity [99]. As a result, they found that the double bond and its *trans*‐configuration are pivotal structural features for the compound's activity since the saturation or inversion of the configuration (*cis* isomer) abolished or reduced the inhibition of snake venom hemorrhagic activity, respectively. Likewise, the carboxylic group was found mandatory for the activity since its methyl ester or aldehyde and alcohol replacements were devoid of any inhibition activity. At odds, the number and positions of the hydroxyl groups were found not critical for the antihemorrhagic activity of rosmarinic acid analogs. Although no structural studies were attempted to solve SVMP crystal structures in complex with **1**, the above SAR scheme pinpoints key pharmacophoric elements that suggest the specificity of rosmarinic acid's mechanism of action for the inhibition of hemorrhagic activity mediated by SVMPs.

In a more recent study, the neutralizing property of the aqueous extract of *Rosmarinus officinalis L*. with a high content of rosmarinic acid (**1**) was proved against the liver toxicity, myotoxicity, and hemorrhagic effects induced by the venom of Egyptian viper *Cerastes cerastes* after its injection in mice [64]. This latter study provides some evidence‐based results supporting the historical sources about the use of plant extracts containing rosmarinic acid (**1**) for the treatment of snakebite envenoming in classical antiquity.

Caffeic acid (**2**) is another natural polyphenol belonging to the chemical group of hydroxycinnamic acids and has been reported to have multiple nutraceutical and pharmacological activities [100]. While it is commonly present in plant‐based foods, it is also contained in many plants used in folk medicine and historical medical treatises to treat snakebite envenoming (Table 1).

The first description of caffeic acid (**2**; Figure 2) as an antidote for the treatment of hemolytic snake venoms is reported in a patent application dating back to the late ‘70s, wherein pharmaceutical preparations containing caffeic acid or analogs and methods of their use were provided for the treatment of snakebite victims [101].

Yet, a recent study has reported that no inhibition activity against the neuromuscular blocking function of PrTX‐I isolated from *Bothops pirajai* can be ascribed to caffeic acid (**2**) and aristolochic acid (**3**), which is another natural compound of folk medicine and historical antidotes (Table 1) with reported antivenom properties [102, 103]. Yet, the same authors were able to prove a partial inhibition of both compounds against the myotoxic activity of PrTX‐I [104]. The lack of neutralizing activity of caffeic acid (**2**) against the snake venom‐induced neuromuscular blockade was also observed in an independent study using an in vitro model of murine phrenic nerve‐diaphragm preparations and a venom mixture collected from *Crotalus durissus terrificus* [105]. Crystallization experiments of caffeic acid (**2**) and aristolochic acid (**3**) bound to PrTX‐I unveiled the presence of multiple binding sites for the former compound and a different single binding site for the latter [104]. Although both compounds interact with residues of the membrane disruption site, one binding site of caffeic acid (**2**) is located close to the entrance of the phospholipid hydrophobic channel of PLA2‐like toxins (Figure 4a,b). This latter site overlaps with that of rosmarinic acid (**1**) in PrTX‐I, suggesting a common mechanism of inhibition of myotoxic activity between rosmarinic acid (**1**) and caffeic acid (**2**).

**FIGURE 4:**
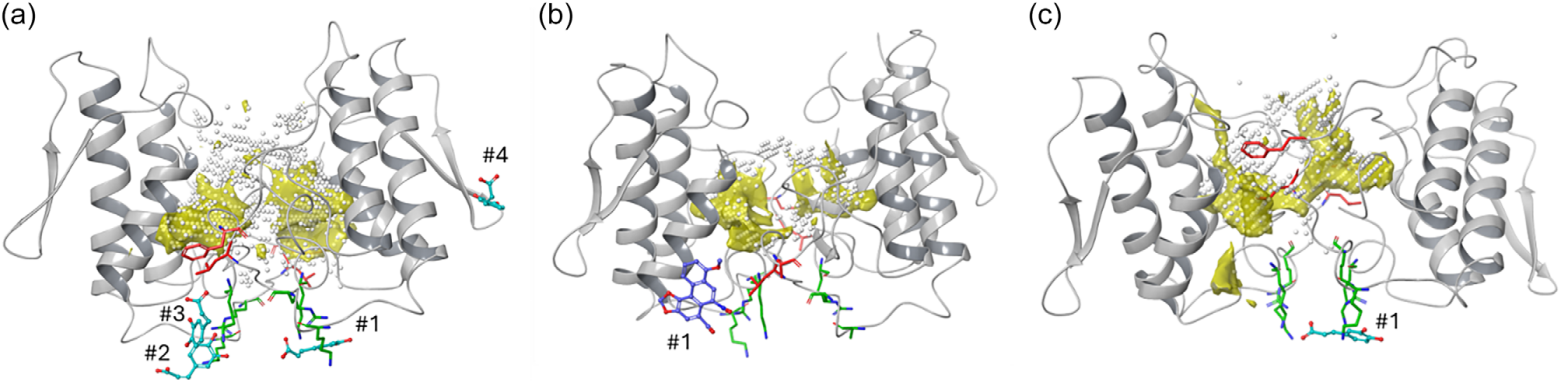
(a) Crystallographic structure of caffeic acid (**2**) bound to PrTX‐I dimer (pdb codes: 4YU7). (b) Crystallographic structure of aristolochic acid (**3**) bound to PrTX‐I dimer (pdb code: 4YZ7). (c) Crystallographic structure of caffeic acid (**2**) bound to BnSP‐7 dimer (pdb codes: 5VFJ). The hydrophobic channel is mapped with the yellow area using SiteMap (Schrodinger 2025, v.14.6); residues of membrane docking site and disruption site are shown in green‐carbon sticks and red sticks, respectively. Caffeic acid and aristolochic acid are shown in cyan‐ and purple‐carbon sticks, respectively, the protein in gray ribbons. Ligand binding sites are numbered.

This finding was partly confirmed in a further study that solved the crystal structure of caffeic acid (**2**) in complex with another PLA2‐like toxin (BnSP‐7) isolated from the *Bothrops pauloensis* venom [106]. In this case, a single binding site of **2** was observed to BnSP‐7 (Figure 4c). This site is exposed to the solvent and located close to the membrane docking site yet far from the hydrophobic channel and membrane disruption site of this PLA2‐like toxin (Figure 4c). The authors suggested that the interaction of caffeic acid (**2**) with this site leads to masking the toxin's membrane docking site, thereby accounting for the inhibition of the protein's myotoxic activity by the compound. To further validate this single caffeic acid binding site, the authors also performed ligand binding experiments using microscale thermophoresis (MST). Results yielded a dissociation constant of caffeic acid (**2**) to BnSP‐7 in the micromolar range of potency (Kd = 20 ± 10 μM).

Chlorogenic acid (**4**; Figure 2) is a polyphenol bearing a hydroxycinnamic chemical scaffold linked to quinic acid. In a comparative study of the antivenom properties of chlorogenic acid (**4**) and rosmarinic acid (**1**) against *Bothrops leucurus* snake venom, researchers found that both compounds were able to inhibit the proteolytic, hyaluronidasic, and procoagulant activities of the homologous PLA2 toxins in vitro, with compound **1** showing more efficacy than **4** [107]. In vivo experiments using mice pinpointed that both inhibitors were able to reduce paw edema induced by the snake venom as well as its hemorrhagic and myotoxic actions. In terms of venom‐mediated systemic toxicity, both compounds proved to mitigate changes in serum biochemical and hemostatic markers while decreasing lipid peroxidation in liver and kidney tissues.

In a more recent study, the molecular interaction of chlorogenic acid (**4**) to BthTX‐I from *Bothrops jararacussu* was investigated combining biological, biophysical, and computational approaches [108].

Chlorogenic acid (**4**) was found to have a protective effect against the muscle damage activity of BthTX‐I with a preincubation treatment while showing partial efficacy against the toxin's neuromuscular blockade activity. Ligand binding experiments with MST confirmed the direct interaction of **4** to BthTX‐I, yielding a Kd of 1.6 ± 0.5 μM. Docking studies were then instrumental to identify the binding site of chlorogenic acid (**4**) at the hydrophobic channel of the active and inactive conformations of BthTX‐I. Notably, molecular dynamic simulations of **4** bound to both the active and inactive conformations of BthTX‐I suggested that the occupancy of the hydrophobic channel by chlorogenic acid stabilizes the inactive conformation, whereas it disrupts the active conformation of the toxin leading to the dissociation of the dimeric assembly.

Although the current interest in compounds **1**‐**4** stems from their use in folk medicines, these molecules also appear as active constituents in some of the medicinal plants used in the preparation of theriac, mithridatum, Orvietan, and scorpion oil (Table 1). However, modern drug development criteria often deem them unattractive because they are broadly active across assays and difficult to optimize [109]. Indeed, these compounds possess at least one catechol group, i.e., a PAINS substructure. Catechol shows a high propensity to redox reactions, can engage metals as a chelating group, and forms a covalent bond in the oxidized form with nucleophilic side chains of protein residues, such as cysteine and lysine [110]. Notwithstanding, some recent works have challenged the reductionistic view of banning PAINS compounds from the practice of lead optimization in medicinal chemistry [111, 112]. These studies advocate for embracing a less restrictive approach based on the understanding of the context of disease and mechanisms of action of compounds. This would indeed be a more appropriate path for a successful snakebite envenoming drug development approach including natural compounds, wherein hitting more than one target could lead to more beneficial outcomes than pursuing the development of selective compounds against one toxin target.

### 3.2 Drug Repurposing

Drug repurposing is an approach that is attracting considerable attention over the last two decades [113]. It consists of reinvestigating approved drugs and/or drug candidates that have not succeeded in clinical trials but are safe for new therapeutic applications. In this framework, identifying known drugs with antiophidian activity represents a viable, low‐cost strategy for discovering novel therapeutic agents against snakebite envenoming [114].

Suramin (**5**; Figure 2) is a polysulfonated compound that was originally developed as one of the first anti‐infective agents for the treatment of African sleeping sickness [115]. Its design and synthesis were accomplished in one of the earliest lead optimization programs by medicinal chemists at Bayer, starting from the trypan blue as a lead compound endowed with antitrypanosomal activity [116]. While the compound is still being used for the treatment of African trypanosomiasis [117] and has been investigated for onchocerciasis, viral infections, cancer diseases, and autism [118, 119, 120], the finding that shows inhibitory activity against mammalian thrombin and PLA2 enzymes [121, 122], prompted researchers to assess its efficacy for the treatment of snakebite envenoming in preclinical studies. It was found that suramin (**5**) can antagonize the neurotoxic and myotoxic effects of some snake venoms in vitro and in vivo, inhibiting the activities of presynaptic neurotoxins and myotoxins, such as crotoxin or β‐bungarotoxin from *Acanthophis* species and the PLA2‐like toxins from *Bothrops* species [123, 124, 125, 126, 127, 128]. Crystallographic studies have provided insights into the binding mode and mechanism of inhibition of suramin (**5**) toward snake venom toxins. Specifically, four crystal structures of the compound bound to PLA2‐like toxins are available, including BaspTX‐II from *Bothrops asper* [126], Ecarpholin S from *Echis carinatus sochureki* [129], and MjTX‐I [130] and MjTX‐II [131] from *Bothrops moojeni*. It was found that suramin (**5**) induces different oligomeric states in PLA2‐like toxins while adopting different binding modes and ligand/protein stoichiometric ratio (Figure 5). Specifically, one suramin molecule binds to BaspTX‐II and MjTX‐I toxins, occupying the hydrophobic channel of each active dimeric complex. At odds, three and two suramin molecules respectively bind to Ecarpholin S and MjTX‐II, interacting with the N‐terminal, C‐terminal, and interfacial recognition residues of the former toxin and residues located near the membrane docking and disruption sites of the latter toxin, establishing electrostatic interactions and hydrophobic contacts. As a result, suramin inhibits these PLA2‐like toxins by sterically blocking the access of membrane phospholipids to the hydrophobic channel (BaspTX‐II, MjTX‐I), perturbing the association of the membrane docking and disrupting sites (Ecarpholin S, MjTX‐II) to the target cells by the negative electrostatic charges of its sulfate groups and eventually by ligand‐induced changes of the quaternary structure of the active dimeric form of the toxins.

**FIGURE 5:**
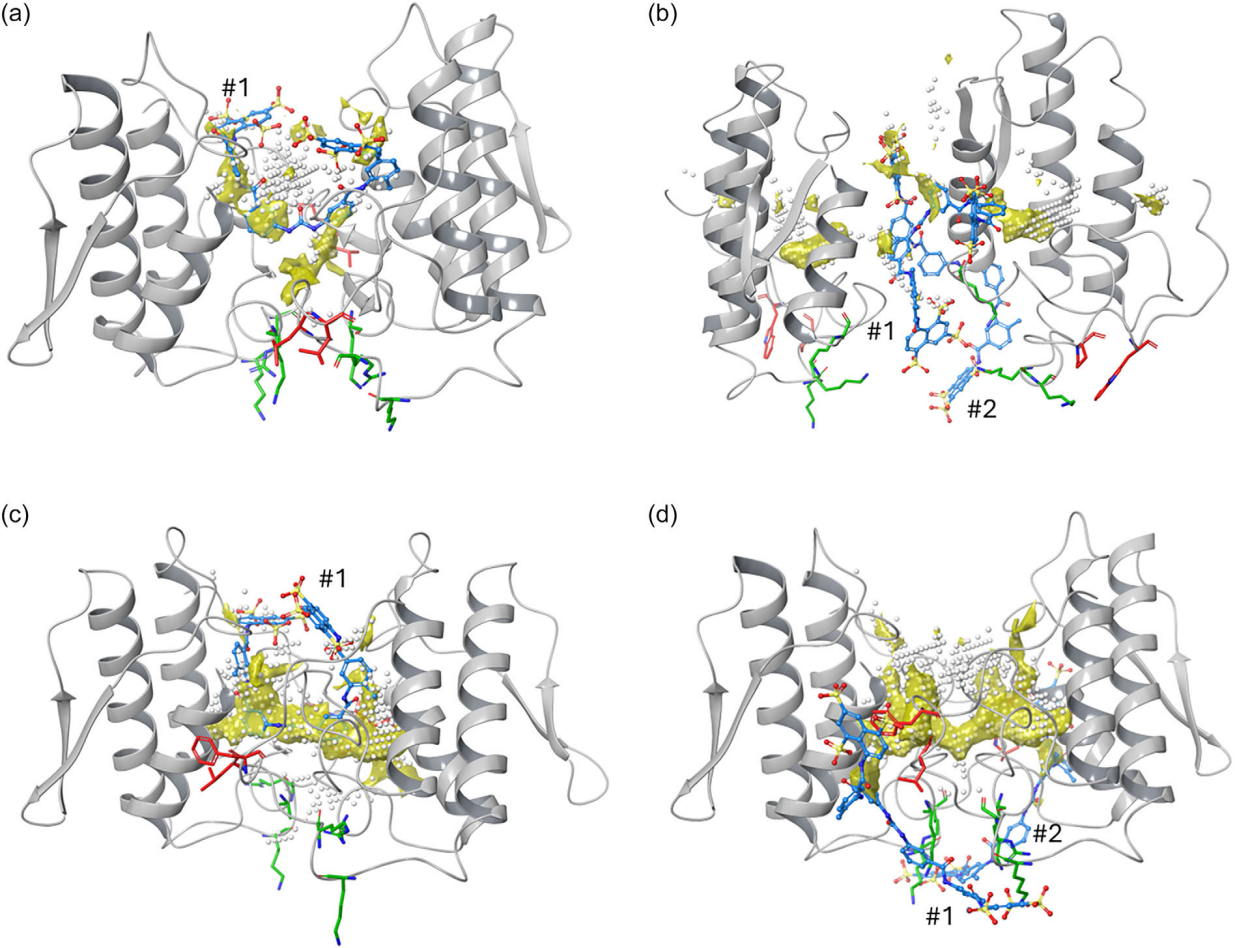
Crystallographic structure of suramin (**5**) bound to BaspTX‐II dimer ((a); pdb code: 1Y4L), Ecarpholin S dimer ((b); pdb code: 3BJW), MjTX‐I dimer ((c); pdb code: 6CE2) and MjTX‐II dimer ((d); pdb code: 4YV5). The hydrophobic channel is mapped with the yellow area using SiteMap (Schrodinger 2025, v.14.6); residues of membrane docking site and disruption site are shown in green‐carbon sticks and red sticks, respectively. Suramin is shown in blue‐carbon sticks and the protein in gray ribbons. Ligand binding sites are numbered.

Unithiol (**6**; Figure 2) is a metal chelator currently used for the treatment of poisoning by heavy metals including arsenic, lead, and mercury. It is administered orally and parenterally, and its mechanism of action consists in the formation of metal complexes, which increase their renal excretion and decrease the concentration of metals in kidneys and other organs. Unithiol was reported to inhibit the activity of Zn^2+^‐dependent SVMPs in vitro, while it was able to prolong or confer complete survival in murine models of snakebite envenoming [132]. Recently, results of a phase 1 study of oral administration of unithiol (**6**) in healthy Kenyan adults for the treatment of snakebite envenoming have been reported in the literature, highlighting its safety, well tolerability, and promising pharmacokinetic properties which pave the way for its development as a rural field‐ready treatment [133].

Marimastat (**7**; Figure 2) is an orally active anticancer drug candidate which was discontinued in phase III clinical studies for the observed mild or no survival benefits. It is a hydroxamic acid derivative that potently inhibits the catalytic activity of matrix metalloproteinases (MMP), which have been involved in destruction of the extracellular matrix and implicated in tumor growth and metastasis. Marimastat (**7**) demonstrated the ability to neutralize the procoagulation effects of viperid venom toxins in a coagulopathic bioassay [134]. It also proved to prolong the survival of mice after snakebite envenomation by intravenous administration. However, a lower efficacy was observed for marimastat (**7**) in inhibiting the local hemodynamic alterations of venom toxins at the site of bite [135]. Yet, no crystallographic studies have been reported that investigate the binding mode of compound **7** to SVMPs. Marimastat (**7**) is currently being studied, as a single oral therapy or in combination with unithiol (**6**) and varespladib (**8**), in phase II clinical trials (TOCSINS: trials of oral combination therapies for snakebite envenoming) by a consortium of organizations from the United Kingdom (UK), Ghana, Kenia, Brazil, and Netherland led by the Liverpool School of Tropical Medicine (UK) [136, 137].

Varespladib (**8**; Figure 2) is a broad‐spectrum inhibitor of secretory PLA2s that was originally developed by Eli Lilly and Shionogi for the treatment of sepsis‐induced systemic inflammatory response syndrome and then licensed to Anthera Pharmaceuticals, which shifted its development as treatment of acute coronary syndrome [138]. However, the developments of this drug candidate and its orally bioavailable prodrug, methyl‐varespladib (**9**), were discontinued for the claimed indications due to disappointing results in advanced clinical settings.

Varespladib (**8**) and methyl‐varespladib experienced a new lease of life when rediscovered as potent inhibitors of snake secretory PLA2‐like proteins and catalytically active PLA2s and thus able to prolong the survival of animal models against lethal doses of venoms from both Elapidae and Viperidae species in preclinical proof‐of‐concept studies [139, 140, 141, 142]. Although these early studies have been thoroughly reviewed elsewhere [139], more recent works have provided further experimental evidence supporting the protective activity of varespladib (**8**) against snake venom toxicity. In a recent study, it was highlighted that dual drug combinations of compound **7** with unithiol (**6**) or marimastat (**7**) were more effective than single therapy in preventing local venom‐induced dermonecrotic damage in mice when coadministered with venoms of *Bothrops asper*, *Crotalus atrox,* and *Echis ocellatus* or even 1 h after the venom challenge [114]. In a further study extending these findings, the same authors investigated the protective effect of varespladib (**8**) in the context of dermonecrosis and myotoxicity induced by spitting cobra (*Naja nigricollis*) envenoming in mice using different routes of drug administration [30]. It was found that the intravenous and intramuscular injections of varespladib (**8**) resulted in significant reductions of myotoxicity, while only the intradermal administration of the drug at the same site of the venom challenge was able to reduce dermonecrosis. While these results question the efficacy of an oral formulation of varespladib (**8**) at preventing local venom‐induced dermonecrosis, likely due to poor peripheral tissue penetration of the systemically administered compound, other studies have explored the protective effects of varespladib (**8**) against systemic venom‐induced toxicity of different snake *genera* and species [143, 144, 145, 146, 147, 148]. Notably, some of these studies have evidenced the protection of varespladib (**8**) against acute liver injury occurring upon *Naja atra* envenoming [141, 142] and its neutralizing effect against the venom anticoagulation activity of two *Bungarus* species (*B. flaviceps* and *B. caeruleus*) [143] and some Australasian elapid snakes [148].

More recently, varespladib (**8**) was used to prepare a therapeutic cocktail against snakebite envenomation, adding in the formulation two neurotoxin (3FTXs)‐targeting antibodies recovered from the memory B cells of a hyperimmune human donor who was exposed to a broad diversity of venoms [149]. The formulation was used in mice to conduct proof‐of‐concept studies of universal antivenom. Results showed complete or partial protection of mice challenged with venoms of 19 different elapid species and genera included in the WHO's list of deadly snakes.

From a structural point of view, crystallographic studies revealed multiple binding sites of varespladib (**8**) into venom PLA2‐like toxins [150, 151, 152]. Specifically, it occupies the interior of the hydrophobic channel in both monomers of MjTX‐I, MjTX‐II, and PrTX‐I while further interacting with the membrane disruption site in one monomer of PrTX‐I (Figure 6). Consequently, a tentative mechanistic model for the myotoxic inhibition of the compound may rely on blocking the entry of membrane phospholipids for toxin activation (MjTX‐II, PrTX‐I), interfering with the association of the membrane docking site (MjTX‐I) to the target cells, masking the membrane disruption site (MjTX‐II, PrTX‐I), and eventually inducing a distorted conformation of the active dimer of the toxin (MjTX‐II), as also observed for rosmarinic acid (**1**) bound to MjTX‐II.

**FIGURE 6:**
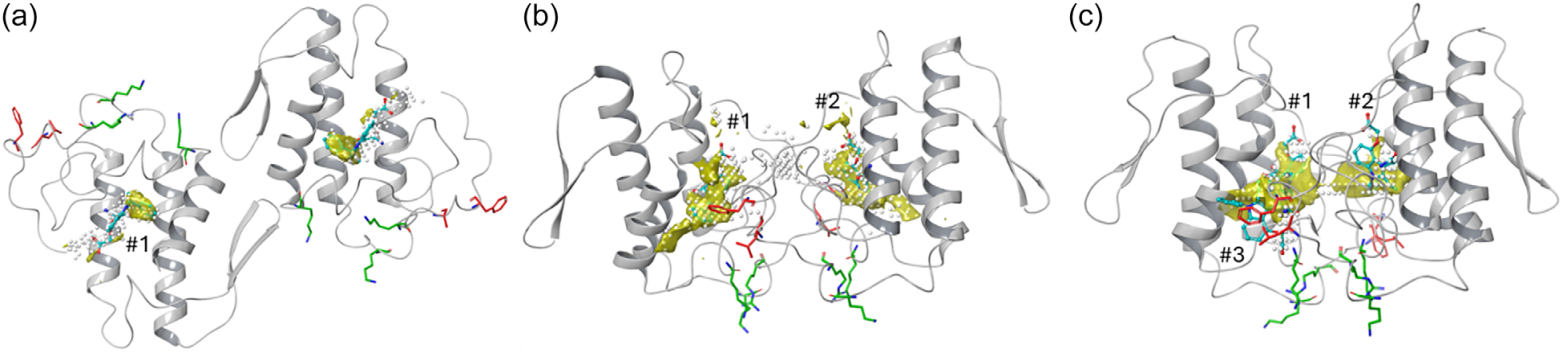
Crystallographic structure of varespladib (**8**) bound to MjTX‐I dimer ((a); pdb code: 7LYE), MjTX‐II dimer ((b); pdb code: 6PWH) and PrTX‐I dimer ((c); pdb code: 8DND). The hydrophobic channel is mapped with the yellow area using SiteMap (Schrodinger 2025, v.14.6); residues of membrane docking site and disruption site are shown in green‐carbon sticks and red sticks, respectively. Varespladib is shown in cyan‐carbon sticks and the protein in gray ribbons. Ligand binding sites are numbered.

Ophirex Inc. has recently started two human trials (BRAVO and BRAVIO) to assess the efficacy of the oral prodrug methyl‐varespladib (**9**) added to standard antivenom therapy against snakebite envenomation. The results of the BRAVO study have been published [153]; although the primary endpoint based on the Snakebite Severity Score (SSS) was not met, signals of clinical benefit were observed in a subgroup of patients initiating the candidate drug therapy within 5 h after the bite. Yet, several factors may have provided confounding effects in the trial primary outcome such as limitations of the adopted SSS measure, delayed initiation of the study drug after the snakebite, concurrent administration of standard antivenom therapy (masking the small molecule efficacy), and heterogeneity in the clinical manifestations of snakebite envenoming symptoms. These limitations have somewhat been recognized by the FDA that agreed to Ophirex Inc. pursuing the development of varespladib for snakebite envenoming under the Animal Rule, thereby permitting the potential approval of the drug for human use using safety data from humans and efficacy data from well‐controlled animal studies.

## 4 Summary and Outlook

Snakebites have long posed a serious threat to human health. While once widespread globally, today, their burden mostly affects countries with limited access to healthcare and medicines. For this reason, the WHO has classified snakebite envenoming as a neglected tropical disease, indicating the supply of treatments to reduce mortality and disability of snakebite envenomation as a strategic objective.

Currently, the only available therapies are indeed animal‐derived polyclonal antibodies, which have major drawbacks, including high production costs, limited cross‐species efficacy, and adverse reactions.

Yet, the quest to find effective antidotes date back to classical antiquity, when complex plant‐ and animal‐based remedies like theriac, mithridatum, and their extended versions produced in the Renaissance like Orvietan or Mattioli's scorpion oil were widely used. Although these historical antidotes were abandoned in most European countries by the 19th century due to lack of experimentally proven efficacy, plant‐based treatments remain common in traditional medicine in India, Asia, and south and central Americas. These practices have inspired growing pharmacological research aimed at identifying natural small molecules with antivenom activity, offering potential alternatives to animal‐derived polyclonal antibodies.

As a result of these efforts, some polyphenols (**1, 2, 4**)—which are also active components of medicinal plants used in theriac, mithridatum, Orvietan, and scorpion oil (Table 1)—have been documented with functional data and structure‐based evidence supporting their specific antiophidian mechanisms of action. Although modern drug development standards would consider these compounds unattractive due to their promiscuous activity in multiple assays, a more context‐aware perspective—particularly in snakebite envenoming, where multitarget effects may be beneficial—would suggest that these compounds may still hold therapeutic value despite their PAINS classification.

More recently, drug repurposing strategies have led to the identification of known drugs (**5**‐**9**), including varespladib (**8**), as promising candidates for the treatment of snakebite envenoming. Notably, some studies have shown improved antivenom efficacy when these compounds were used in combination—either with one another or in conjunction with human antibodies—providing a clue that the complex herbal mixtures found in historical antidotes might have been perhaps effective (or at least in part) because they contained compounds (e.g., polyphenols) capable of engaging multiple targets and mechanisms of action simultaneously.

Although varespladib (**8**) has recently received FDA Orphan Drug, Rare Pediatric Disease, and Fast Track designations and is being developed as a standalone therapy, further studies are needed to determine whether combination treatments involving also natural compounds and/or human antibodies could provide synergistic effects and broader cross‐species efficacy against both Elapidae and Viperidae venoms. Such a universal antivenom would represent a breakthrough, offering a single therapeutic product capable of protecting against numerous venomous snake species across diverse geographic regions for which no adequate antivenom currently exists.

## Conflicts of Interest

The authors declare no conflicts of interest.

## Data Availability

Data sharing is not applicable to this article as no datasets were generated or analyzed during the current study.
